# Disrupting Hedgehog signaling in melanocytes by SUFU knockout leads to ocular melanocytosis and anterior segment malformation

**DOI:** 10.1242/dmm.050210

**Published:** 2023-08-29

**Authors:** Weizhuo Wang, Feiyang Li, Jing Wang, Zuimeng Liu, Meiyu Tian, Zhenhang Wang, Huirong Li, Jia Qu, Yu Chen, Ling Hou

**Affiliations:** ^1^ Laboratory of Developmental Cell Biology and Disease, State Key Laboratory of Ophthalmology, Optometry and Visual Science, Eye Hospital, Wenzhou Medical University, Wenzhou 325027, China; ^2^ National Clinical Research Center for Ocular Diseases, Eye Hospital, Wenzhou Medical University, Wenzhou 325027, China

**Keywords:** Neural crest, Neurocristopathy, Melanoblast subpopulations, MITF, Pigment cells, Cell migration

## Abstract

Hedgehog (Hh) signaling is well known for its crucial role during development, but its specific role in individual cell lineages is less well characterized. Here, we disrupted Hh signaling specifically in melanocytes by using Cre-mediated cell-type-specific knockout of the Hh regulator suppressor of fused (*Sufu*). Interestingly, corresponding mice were fully pigmented and showed no developmental alterations in melanocyte numbers or distribution in skin and hair follicles. However, there were ectopic melanoblasts visible in the anterior chamber of the eye that eventually displayed severe malformation. Choroidal melanocytes remained unaltered. Surprisingly, the abnormal accumulation of anterior uveal melanoblasts was not the result of increased cell proliferation but of increased migration to ectopic locations such as the cornea. In melanoblasts *in vitro*, *Sufu* knockdown replicated the increase in cell migration without affecting proliferation and was mediated by an increased level of phosphorylated-ERK brought about by a reduction in the levels of the repressor form of GLI3. These results highlight the developmental divergence of distinct melanocyte subpopulations and may shed light on the pathogenesis of human ocular melanocytosis.

## Teaser

**Summary:** *Sufu* deletion in the melanocyte lineage selectively interrupts ocular melanocyte development, leading to ocular melanocytosis and malformation of the anterior segment of the eye.

## INTRODUCTION

Migration of cells to the proper location throughout the body during early development is a fundamental process of normal tissue formation and physiological function. The neural crest (NC) is a unique vertebrate tissue as its cells migrate extensively and contribute to the formation of a number of different organs (Bronner and LeDouarin, 2012; Etchevers et al., 2019). NC cells arise from the dorsal neural tube early in development and migrate along defined pathways through multiple microenvironments to their final destinations. These include the eyes, ears, teeth, bone and cartilage of the head, gut and others (Bronner and LeDouarin, 2012; Etchevers et al., 2019). Understanding NC development and migration is medically important because human birth defects, so-called neurocristopathies, arise from defects in NC cell development often due to gene mutations (Bolande, 1997). To name but a few, mutations in *Sox10* and *Ednrb* cause defects in enteric NC cell migration and hence enteric neurons, leading to megacolon (Lake and Heuckeroth, 2013). Mutations in *Ednrb*, along with mutations in *Kit*, also affect the migration of NC-derived melanoblasts, leading to white spotting in mice and Waardenburg syndrome or piebaldism in humans (Hou and Pavan, 2008; Lee et al., 2003; Wehrle-Haller et al., 2001). Mutations in the *TCOF1* gene lead to a reduction in the number of cranial NC cells, resulting in Treacher Collins syndrome (Masotti et al., 2009). Finally, of particular importance for the present study, improper migration of NC cells in the developing eye has been implicated in anterior segment dysgenesis syndromes (Cavodeassi et al., 2019). Hence, proper NC cell migration is critical to maintain tissue homeostasis and establish proper morphogenesis and function.

The differentiated derivatives of NC-derived melanoblasts, the melanocytes, are primarily known for their melanin pigment, which provides coloration of the skin. In addition, the melanin pigment can protect against the damaging effects of ultraviolet radiation (Abdel-Malek et al., 2010; Baxter et al., 2004; Kapp et al., 2018; Perlis and Herlyn, 2004; Slominski et al., 2022b). There is increasing evidence that melanocytes are not simply melanin-producing cells but have many functions; for instance, skin melanocytes can regulate and maintain body homeostasis. (Slominski et al., 2004, 2022a). Moreover, melanocyte properties are also hormonally regulated, such as by melanocyte-stimulating hormone (MSH), adrenocorticotropic hormone [ACTH; also known as proopiomelanocortin (POMC)], melatonin and serum histamine at local and systemic levels. In the eye, the light-blocking effect of melanin of iris and choroidal melanocytes serves to regulate the amount of light entering the eye and prevent light from reaching deeper structures behind the retina. This latter function is further supported by melanin in a specialized single layer that is not of NC but of brain origin, the retinal pigment epithelium (RPE). There is increasing evidence that NC-derived ocular melanocytes play pivotal roles in ocular homeostasis. For instance, melanocytes produce a key regulator of angiogenesis, fibromodulin, promote angiogenesis in tissues such as the iris and choroid (Adini et al., 2014), and contribute to the maintenance of the choroidal vasculature (Shibuya et al., 2018). Furthermore, the multiple functions of RPE cells in retinal homeostasis have recently been reviewed (Lakkaraju et al., 2020; Ma et al., 2019).

The generation and function of NC-derived melanoblasts and, hence, melanocytes, critically depend on many additional signaling pathways and transcription factors besides the ones already mentioned, such as WNT signaling, MITF and PAX3 (Goding and Arnheiter, 2019; Hou et al., 2006; Larue and Delmas, 2006; Li and Hou, 2018; Saldana-Caboverde and Kos, 2010; Sommer, 2005; Wehrle-Haller, 2003). Melanocytes are also affected by Hedgehog (Hh) signaling, which is a developmentally important signaling pathway, but which has not yet been extensively studied with respect to its role in melanocytes. Hh signaling is evolutionarily conserved and plays an essential role in embryonic development and tumorigenesis (Humke et al., 2010; Ingham et al., 2011; Smelkinson, 2017; Stecca et al., 2007). The pathway is activated by binding of appropriate ligands to the patched 1 (PTCH1) receptor, which leads to activation of the transmembrane G protein-coupled receptor smoothened (SMO). SMO activation ultimately results in the regulation of the transcriptional activity of the glioma-associated oncogene homolog (GLI) proteins, which are zinc finger transcription factors (Mullor et al., 2002). Hh signaling from the midline promotes the segregation of the single eye field into two optic primordia and is required for the correct proximodistal and dorsoventral patterning of the optic vesicle (Bakrania et al., 2010; Cardozo et al., 2014). Mutations in effectors of this signaling pathway in NC cells can cause ocular deformities, including iris hypoplasia, iris coloboma, corectopia and polycoria appearing in Axenfeld–Rieger syndrome, basal cell nevus syndrome and Curry–Jones syndrome (Cavodeassi et al., 2019). It has been shown that melanocytes indeed express key factors of Hh signaling (Alexaki et al., 2010; Stecca et al., 2007). In an elegant sensitized mutagenesis screen, GLI3 has been identified as a modifier of SOX10, the above-mentioned transcription factor involved in NC-derived lineage development, and loss of GLI3 interrupts melanoblast specification (Matera et al., 2008). Nevertheless, how Hh signaling is involved in NC-derived melanocyte development and function remains largely unknown.

Suppressor of fused (*Sufu*) is an inhibitor of the vertebrate Hh signaling pathway and also a negative regulator of WNT/β-catenin and other signaling pathways. It is not only a regulator of cell proliferation, migration and tissue homeostasis, but also prevents the occurrence of malignant tumors (Coquenlorge et al., 2019; Liu et al., 2014; Yung et al., 2019). SUFU regulates the stability, localization and activities of the Hh effectors GLI1, GLI2 and GLI3 (Ruiz i Altaba et al., 2002). Both GLI2 and GLI3 are dual-function transcription factors, containing a C-terminal activation domain and an N-terminal arresting domain; they act as activators when full length (GLI-F) or as repressors when truncated (GLI-R). GLI1, in contrast, can only act as an activator as it lacks the N-terminal arresting domain (Ingham et al., 2011). SUFU can interact with GLI activators as well as repressors, suggesting that it exerts a key function in regulating the levels of Hh signaling. *Sufu-*deficient mouse embryos exhibit aberrant Hh activity, which causes severe cephalic and cardiac malformations and results in embryonic lethality around embryonic day (E)9.5 (Cooper et al., 2005; Svärd et al., 2006). Hence, investigations of the role of SUFU in development require tissue-specific mutational models. Indeed, conditional knockout (cKO) of *Sufu* in NC cells leads to aberrant enteric NC cell migration and disorganization of the enteric nervous system (Liu et al., 2015). In addition, conditional ablation of *Sufu* in cranial NC cells causes failure in calvarial bone formation and significantly inhibits the proliferation and differentiation of osteoprogenitor cells. These failures are due to the dysregulation of BMP and Notch signaling through GLI2-F and GLI3-R (Li et al., 2017b). The early death of these mutants, however, prevents the analysis of the role of SUFU in the development of the melanocyte lineage into adulthood.

In the present study, we specifically addressed the question of the role of Hh signaling in the development of NC-derived melanocytes by conditionally knocking out *Sufu* in the melanocyte lineage using transgenic mice expressing Cre recombinase under the control of the melanocyte-specific tyrosinase promoter (*Tyr-Cre*). We found that *Sufu* deletion does not discernibly affect melanocytes in the skin but leads to abnormal migration of ocular melanoblasts and malformation of the anterior segment in eyes. We further explored the underlying *Sufu*-dependent mechanisms of the regulation of melanoblast migration *in vitro* in a melanoblast cell line, melb-a. The results suggest that *Sufu* regulates melanoblast migration by modulating the expression of phosphorylated (p)-ERK (also known as p-MAPK) and GLI3-R. Hence, it appears that the Hh regulator *Sufu* serves to maintain the normal development of ocular melanoblasts and proper formation of the anterior segment of the mouse eye.

## RESULTS

### Loss of SUFU in the melanocyte lineage leads to ectopic ocular melanocytes and anterior segment malformation

To investigate whether Hh signaling plays any role in the melanocyte lineage, we used the Cre-LoxP system to cell-type-specifically knock out the Hh signaling repressor *Sufu.* To begin with, we examined the specificity and expression efficiency of *Tyr-Cre* in melanocytes. To this end, we constructed the *B6-G/R; Tyr-Cre* mouse as schematically shown in Fig. S1A,B. As shown in Fig. S1C, triple immunostaining for ZsGreen, TdTomato and MITF, the latter a marker for pigment cells, showed that the majority of melanocytes (which are MITF positive) colocalized with TdTomato fluorescence, indicating CRE expression. Then, we crossed *Sufu^f/f^* mice, in which LoxP sites are inserted upstream and downstream of *Sufu* exon 7, with *Tyr-Cre* transgenic mice, in which Cre is expressed in melanoblasts and melanocytes (Fig. S2A,B). As shown in Fig. S2C,D, SUFU was efficiently knocked out in melanocytes at postnatal day (P)6.

To examine whether the loss of *Sufu* in the melanocyte lineage generally affects melanocyte differentiation and pigmentation, we first compared the extent of hair pigmentation, the number and pigmentation of melanocytes in the skin, and the skin's overall morphology between 2-month-old control (*Sufu^f/f^*) and *Sufu*-cKO (*Sufu^f/f^; Tyr-Cre*) mice. Surprisingly, the specific knockout of *Sufu* in the melanocyte lineage did not cause any detectable abnormalities in the color or structure of dorsal or ventral hair (Fig. S3A,B) or hairs from the tail or paw (Fig. S3C,D). There were also no significant color abnormalities in facial or body skin after epilation (Fig. S3E,F). As shown in Fig. S3G, there were no histological differences in skin, ears, tails and paws between knockout and wild-type mice. To monitor hair growth and pigmentation, we depilated the hair of mice at P21 to induce skin hair follicles in telogen to enter the anagen phase (Fig. S3H). The results showed that there were no differences in pigmentation in hair follicles between *Sufu^f/f^* and *Sufu*-cKO mice at day 3 (P24), day 5 (P26) and day 8 (P29) after depilation. These observations suggested that SUFU is not required for the generation of NC melanoblasts/melanocytes or for the homeostasis of the skin. However, a closer inspection of the eyes showed reduced pigmentation of the RPE, a finding that will be analyzed separately in the future. These data indicate that, under normal physiological conditions, knockout of *Sufu* in the melanocyte lineage does not cause any detectable changes in color, pigmentation or structure of the hair or skin.

**Fig. 1.**
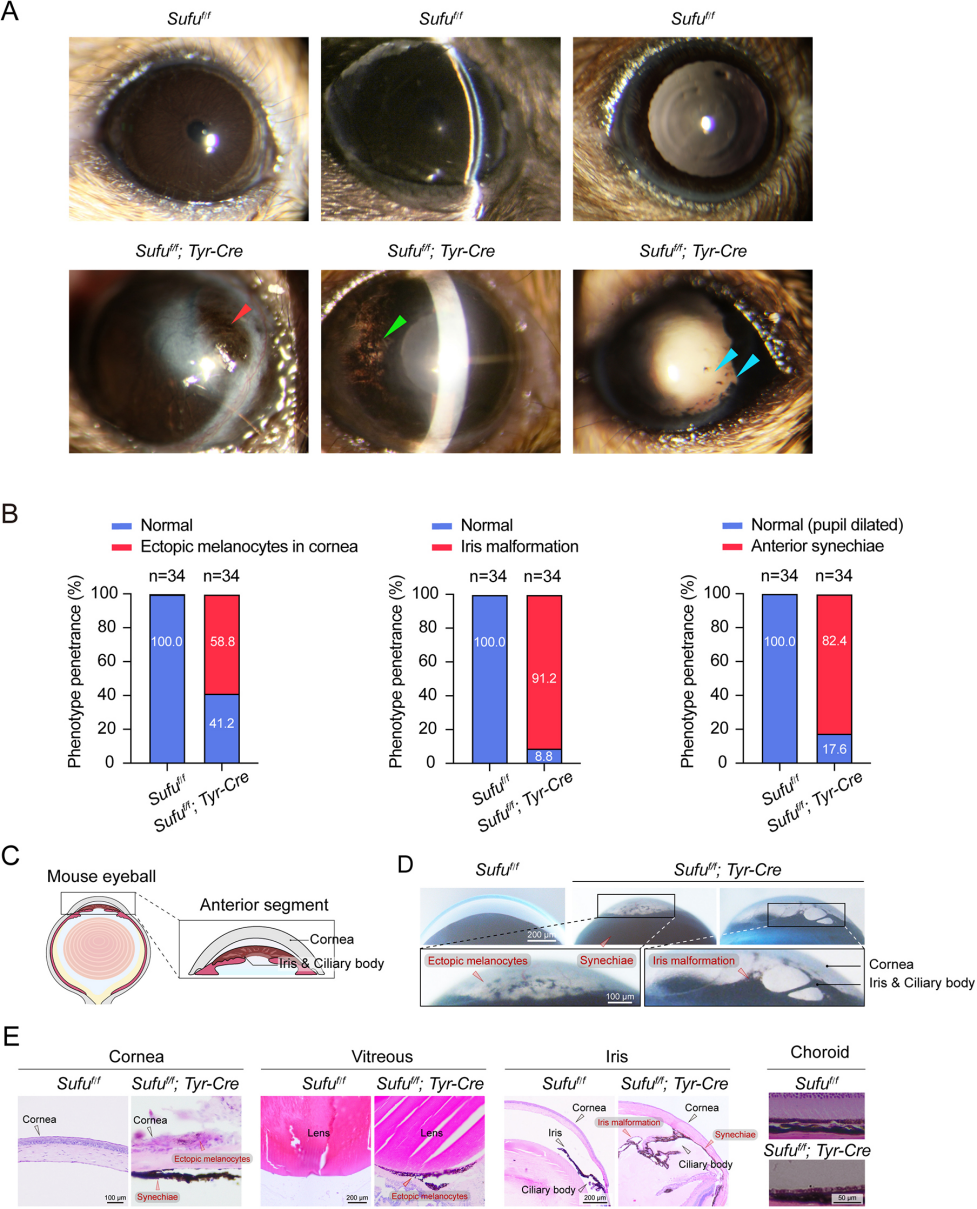
**Conditional knockout (cKO) of *Sufu* in the melanocyte lineage leads to ectopic ocular melanocytes and anterior segment malformation.** All mice used for these studies were 2 months of age. (A) Representative slit lamp microscopic images in *Sufu^f/f^* (control) and *Sufu*-cKO (*Sufu^f/f^; Tyr-Cre*) mice. Note ectopic melanocytes and abnormal anterior segment structures in *Sufu*-cKO mice. Left column: red arrowhead indicates a cluster of melanocytes in the cornea; middle column: green arrowhead indicates the abnormal iris with partial loss of pigment; right column: blue arrowheads indicate that the iris did not dilate sufficiently and some melanocytes adhered to the cornea after mydriatic treatment. (B) Statistical analysis of phenotypes based on the results in A (*n*=34). (C) Schematic drawings of wild-type mouse eyeball and anterior segment. (D) Details of the anterior segment in *Sufu^f/f^* and *Sufu*-cKO mice. Black boxes mark areas with melanocytes adhering to the cornea and iris malformation with abnormal structure. (E) Representative H&E staining images showing ectopic melanocytes and iris malformation. Note that some ectopic melanocytes appeared in the cornea and the iris adhered to the cornea with abnormal morphology. No detectable abnormalities were seen in the choroid of *Sufu*-cKO mice.

As found for the integument, knockout of *Sufu* did also not disrupt the generation of NC melanocytes in the eye but it disrupted their homeostasis in the anterior eye segment. As shown by slit lamp microscopy in 2-month-old mice, a large proportion of the *Sufu*-cKO mice had malformation of anterior segment structures (Fig. 1A,B). Melanocytes appeared in the cornea in 58.8% of *Sufu*-cKO mice*.* Furthermore, 91.2% of *Sufu*-cKO mice showed irregular iris malformation and 82.4% showed iris synechiae after dilation of the pupil. We further examined the anterior segment abnormalities by stereo microscopy. A schematic diagram of the anterior segment structure is provided in Fig. 1C; Fig. 1D shows the many ectopic melanocytes in the cornea and adherence of the iris to the cornea. In addition, *Sufu*-cKO irises displayed irregular holes. Hematoxylin and Eosin (H&E) staining showed ectopic melanocytes in the cornea and malformation of the iris but no obvious abnormalities of the choroid (Fig. 1E). These results indicate that SUFU is required for normal melanocyte positioning in the anterior segment and for proper anterior segment formation and function.

### Loss of SUFU in the melanocyte lineage causes aberrant localization of melanoblasts

**Fig. 2.**
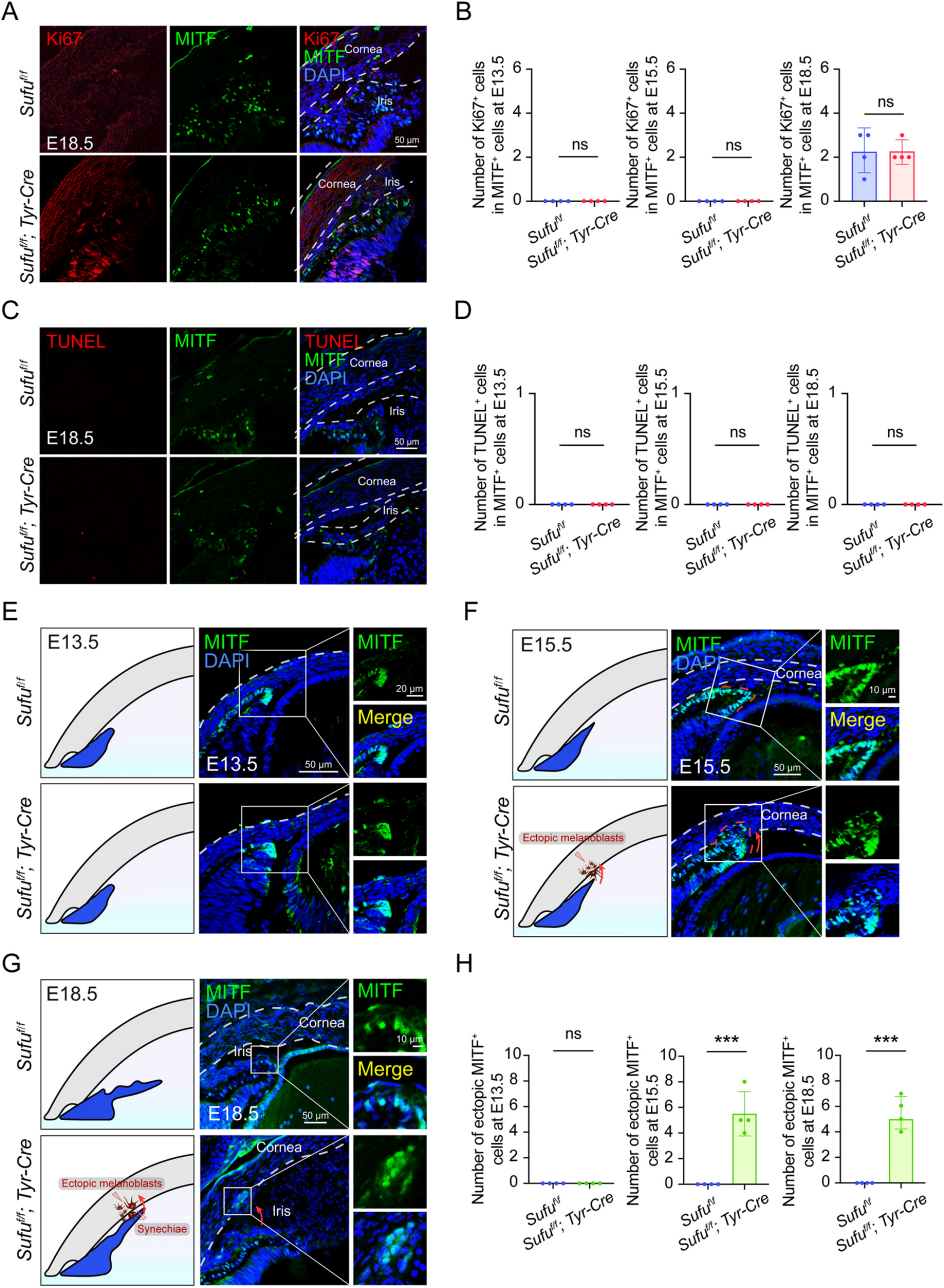
**Loss of *Sufu* in the melanocyte lineage leads to abnormal localization of MITF-positive melanoblasts in developing eyes.** (A) Representative immunofluorescence images of a proliferating cell marker (Ki67; red) in MITF-positive cells (green) of E18.5 embryos of the indicated genotypes. (B) Statistical analysis of double Ki67/MITF-positive cells at E13.5, E15.5 and E18.5 (*n*=4 for each genotype). Note that there were no significant differences between *Sufu^f/f^* and *Sufu*-cKO embryos at the different stages. (C) Representative immunostaining images of an apoptosis cell marker (TUNEL staining; red) in MITF-positive cells (green) of E18.5 embryos of the indicated genotypes. (D) Statistical analysis of double TUNEL/MITF-positive cells at the different stages (*n*=4 for each genotype). (E) Representative immunostaining images of MITF (green) in E13.5 *Sufu^f/f^* and *Sufu*-cKO eyes. Left column: schematic drawings of the anterior segment according to genotype. Right column and insets: MITF-positive melanoblasts (green) of the anterior area of the ciliary margin zone (CMZ). Note the absence of ectopic MITF-positive cells (green) in E13.5 *Sufu*-cKO eyes. (F) Representative immunostaining images of MITF (green) in E15.5 *Sufu^f/f^* and *Sufu*-cKO eyes. Left column: schematic drawings of the anterior segment according to genotype. Right column and insets: in contrast to control *Sufu^f/f^* eyes, *Sufu*-cKO eyes show ectopic MITF-positive melanoblasts (green) associated with, and inserted into, the cornea. The white boxes are shown enlarged in the insets, highlighting the contrast between normally positioned melanoblasts (in *Sufu^f/f^* control) and ectopic melanoblasts (in *Sufu*-cKO). (G) Equivalent schematic and immunostaining images of E18.5 eyes. (H) Statistical analyses of the number of ectopic melanoblasts based on the results in E-G (*n*=4 for each genotype). ***P<0.001; ns, no significant difference (unpaired two-tailed Student's *t*-test).

To explore the potential reason for the ectopic localization of melanocytes and the abnormal anterior segment formation in adult *Sufu*-cKO mice, we first examined ocular samples at E13.5, E15.5 and E18.5, the critical period during which NC-derived melanoblasts reach the anterior area of the ciliary margin zone (CMZ). As a melanoblast-specific marker, we chose MITF, a critical regulator of the melanocyte lineage (Goding and Arnheiter, 2019; Hodgkinson et al., 1993; Hou et al., 2000). As shown in Fig. 2, double immunostaining for MITF and Ki67 (also known as MKI67), the latter a marker for cell proliferation, showed no significant difference in melanoblast proliferation between *Sufu*-cKO and control eyes (Fig. 2A for immunolabeling at E18.5; Fig. 2B for quantitation at E13.5, E15.5 and E18.5). Furthermore, there was also no difference in terminal deoxynucleotidyl transferase-mediated dUTP nick-end labeling (TUNEL)/MITF double-positive cells (Fig. 2C,D; Fig. S4). These results indicate that the cKO of *Sufu* in the melanocyte lineage did not affect the proliferation or apoptosis of melanoblasts in developing eyes, and so cannot account for the differential accumulation of melanoblasts in the anterior segment.

Further examination of the development of the anterior segment and the distribution of melanoblasts revealed the first changes between *Sufu*-cKO and control eyes to emerge at E15.5, when ectopic melanoblasts invading the cornea at the level of the anterior portion of the developing iris appeared (Fig. 2E-H). At E18.5, the anterior iris segment and adjacent cornea of *Sufu*-cKO eyes exhibited many ectopic melanoblasts, and there were partial adhesions between the iris and cornea. In contrast, in control eyes, the developing iris was clearly separated from the cornea, there were no anterior synechiae or adhesions, and MITF-positive melanoblasts were only localized in the iris (Fig. 2G,H). Further, the eyes of E18.5 embryos were examined by stereo microscopy and H&E staining. The pupils of *Sufu^f/f^* embryos were round and smooth (blue arrowhead in Fig. S5A), whereas those of *Sufu*-cKO embryos were irregularly shaped (red arrowhead in Fig. S5A), indicating iris abnormalities. Corresponding histological analysis of the developing eye showed an abnormal iris and ciliary body in *Sufu*-cKO embryos (Fig. S5B).

Collectively, the above results showed that the specific knockout of *Sufu* in the melanocyte lineage did not affect the proliferation or apoptosis of melanoblasts in the anterior eye segment but resulted in abnormal localization of melanoblasts starting around E15.5. This abnormal melanoblast localization was associated with disruption of the normal formation of the anterior segment. Hence, the normal morphogenesis of the anterior segment of eyes requires SUFU in the ocular melanocyte lineage.

### SUFU regulates melanoblast migration by modulating MAPK signaling

**Fig. 3.**
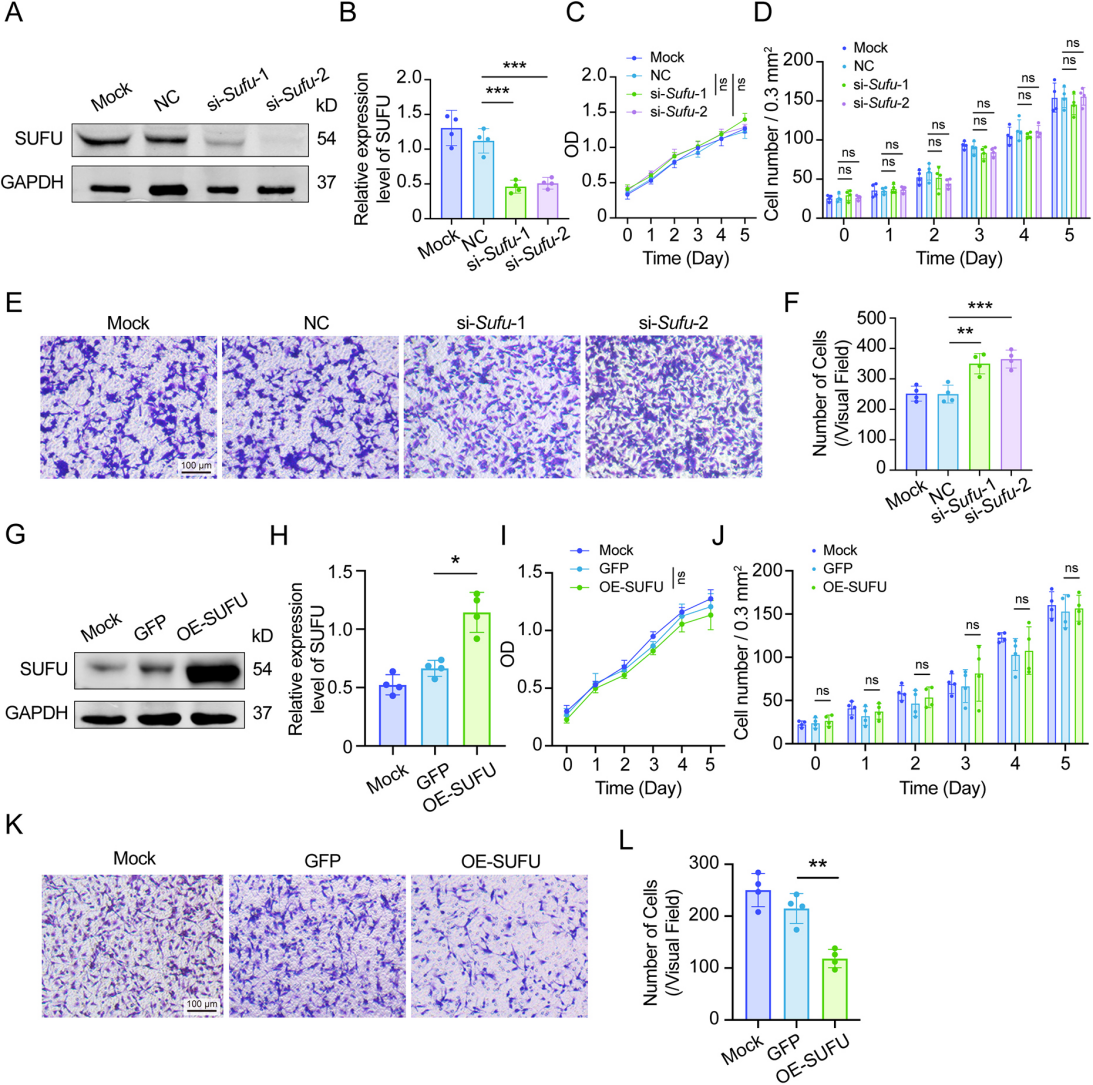
***Sufu* regulates the migratory capacity of melanoblasts *in vitro*.** (A) Expression levels of SUFU in a melanoblast cell line, melb-a, when transfected with si-*Sufu*-1 or si-*Sufu*-2 (*n*=4). (B) Statistical analysis of western blots based on the results in A. (C) Growth curve of melb-a cells after mock, si-NC, si-*Sufu*-1 or si-*Sufu*-2 transfection as measured by CCK-8 (*n*=4). (D) Number of melb-a cells after mock, si-NC, si-*Sufu*-1 or si-*Sufu*-2 transfection by cell counting (*n*=4). Note the absence of any differences in cell counts. (E) Cell migration as evaluated in a transwell migration assay after mock, si-NC, si-*Sufu*-1 or si-*Sufu*-2 transfection (*n*=4). Note that knockdown of *Sufu* promoted melb-a cell migration. (F) Statistical analysis of migration based on the results in E. (G) Expression levels of SUFU in melb-a cells after lentivirus-SUFU infection (*n*=4). (H) Statistical analysis based on the results in G. (I) Growth curve of melb-a infected with GFP or OE-SUFU lentivirus as measured by CCK-8 (*n*=4). (J) Number of melb-a cells infected with GFP and OE-SUFU lentivirus detected by cell counting (*n*=4). Note that the growth curve and cell counting showed no significant difference in cell numbers after upregulating *Sufu*. (K) Cell migration as evaluated in a transwell assay after overexpression of SUFU by lentivirus-SUFU infection. Note that overexpression of SUFU inhibited melb-a cell migration (*n*=4). (L) Statistical analysis based on the results in K. OD, optical density; OE-SUFU, overexpression of SUFU. *P<0.05, **P<0.01, ***P<0.001; ns, no significant difference (one-way ANOVA).

Ectopic localization of melanoblasts could be due to aberrant retention at anatomical sites normally traversed by developing cells or aberrant migration to sites not normally occupied by melanoblasts. We reasoned that distinguishing between these possibilities and exploring the underlying molecular mechanisms may be difficult *in vivo*; hence, we switched to a more feasible *in vitro* system. To this end, we used a mouse melanoblast cell line, melb-a, in which we manipulated the levels of functional SUFU. First, we used siRNAs to knock down the expression of *Sufu*. Transfection of melb-a cells with either si-*Sufu*-1 or si-*Sufu*-2 led to significantly lower expression levels of SUFU protein compared to mock or control siRNA-transfected cells (Fig. 3A,B). Next, we used CCK-8 and cell counting to assess cell proliferation. Consistent with *in vivo* results, the growth curve was not affected by downregulating *Sufu* (Fig. 3C,D). We then performed a transwell migration assay to measure the migratory capacity of cells. As shown in Fig. 3E and F, knockdown of *Sufu* led to increased cell migration compared to that in mock or control siRNA-treated cells. Likewise, overexpression of SUFU by lentivirus-SUFU (Fig. 3G,H) did not significantly alter the proliferative capacity of melb-a cells compared to that of mock infected cells or cells overexpressing a control protein (GFP) (Fig. 3I,J). However, SUFU overexpression led to reduced migration of melb-a cells in transwell assays (Fig. 3K,L). These data indicate that the downregulation of SUFU promotes, and its upregulation inhibits, the migratory capacity of melanoblasts without affecting cell proliferation.

**Fig. 4.**
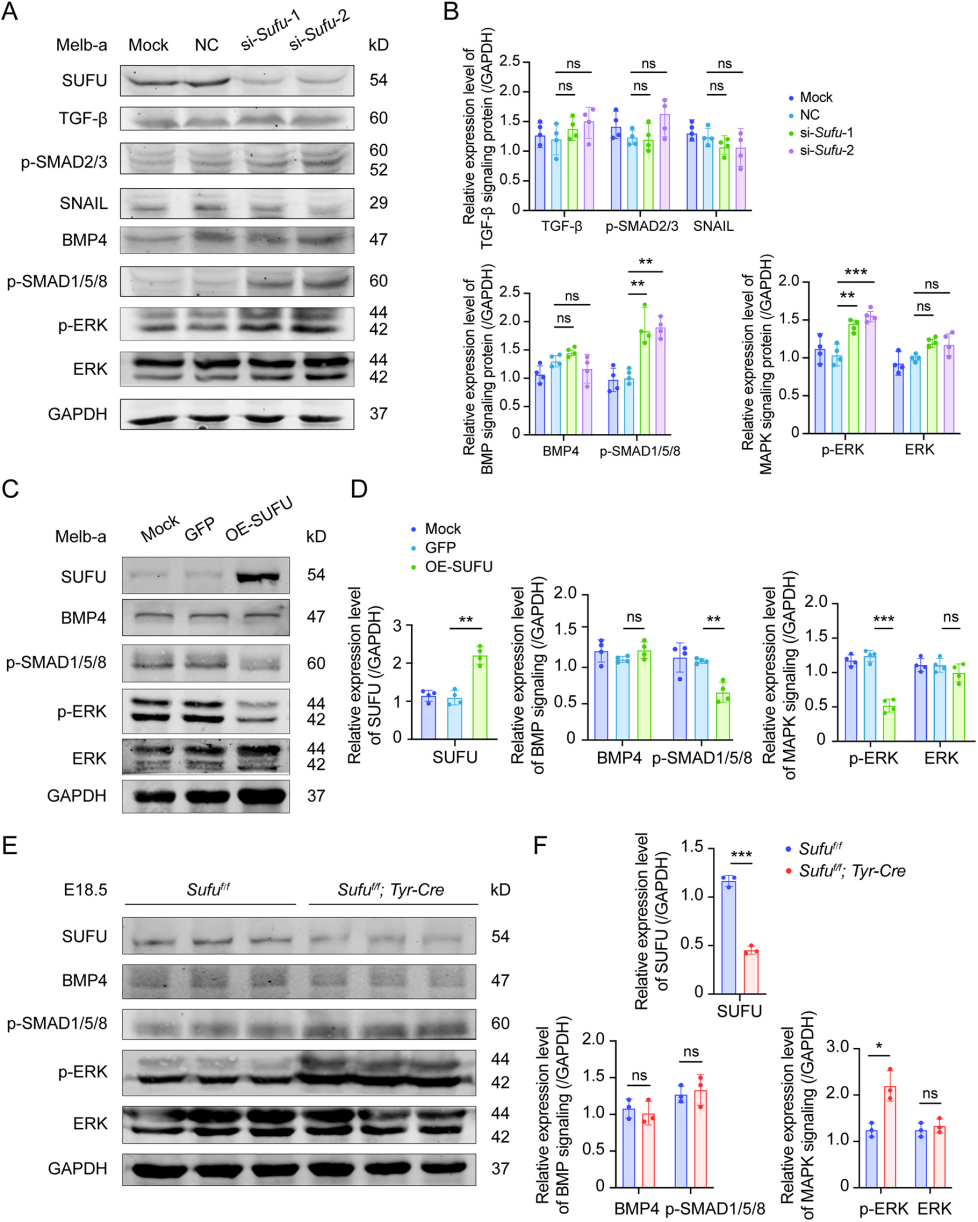
**The expression levels of critical proteins in TGF-β, BMP and MAPK signaling pathways are associated with cell migration.** (A) Expression levels of members of the TGF-β, BMP and MAPK signaling pathways in melb-a cells after mock, si-NC, si-*Sufu*-1 or si-*Sufu*-2 transfection. Note that there were no significant differences in TGF-β signaling after knockdown of *Sufu*, but BMP signaling and MAPK signaling were upregulated (*n*=4). (B) Statistical analysis of signaling-related protein expression based on the results in A. (C) Expression levels of critical BMP and MAPK signaling pathway components after lentivirus-SUFU infection of melb-a cells. Note that both p-SMAD1/5/8 and p-ERK levels were downregulated when SUFU was overexpressed, whereas BMP4 and overall ERK levels were not (*n*=4). (D) Statistical analysis of signaling-related protein expression based on the results in C. (E) *In vivo* expression levels of critical proteins of the BMP and MAPK signaling pathways in the iris and ciliary body (both rich in melanoblasts) as assayed in E18.5 *Sufu^f/f^* and *Sufu*-cKO eyes. Note that there were no significant differences in BMP signaling after knockout of *Sufu*, but MAPK signaling was upregulated (*n*=4). (F) Statistical analysis of protein expression based on the results in E. *P<0.05, **P<0.01, ***P<0.001; ns, no significant difference (two groups, unpaired two-tailed Student's *t*-test; multiple groups, one-way ANOVA).

The regulation of cell migration has previously been linked to the TGF-β, BMP and MAPK signaling pathways (Li et al., 2011; Liou et al., 2002; Liu et al., 2009; McBain et al., 2003). Hence, we examined whether *Sufu* knockdown would lead to changes in distinct ligands or effector proteins of these pathways. As shown in Fig. 4A, there were no significant differences in TGF-β, p-SMAD2/3, SNAIL (also known as SNAI) or BMP4. However, the protein levels of p-SMAD1/5/8, the crucial transcriptional effectors of BMP signaling, were upregulated after si-*Sufu*-1 or si-*Sufu*-2 transfection. Likewise, the expression levels of p-ERK, the essential effector of MAPK signaling, were also increased, whereas overall ERK levels were unchanged (Fig. 4A,B). Conversely, overexpression of SUFU led to a decrease in p-SMAD1/5/8 expression and also downregulated p-ERK, without changing overall ERK levels (Fig. 4C,D).

To confirm these observations *in vivo*, we isolated the iris and ciliary body (both rich in melanoblasts) from E18.5 wild-type and *Sufu*-cKO embryos. Because the above results gave no indication of changes in the TGF-β signaling pathway, we focused on BMP4 and MAPK signaling. In contrast to the *in vitro* results, western blot analysis showed no significant changes in p-SMAD1/5/8 in *Sufu*-cKO versus control tissue. The expression of p-ERK, however, was significantly upregulated, without changes in the overall ERK levels (Fig. 4E,F). These results suggest that *Sufu* significantly modulates MAPK signaling to regulate the migratory capacity of melanoblasts.

### SUFU works through MAPK signaling to regulate melanoblast migration

**Fig. 5.**
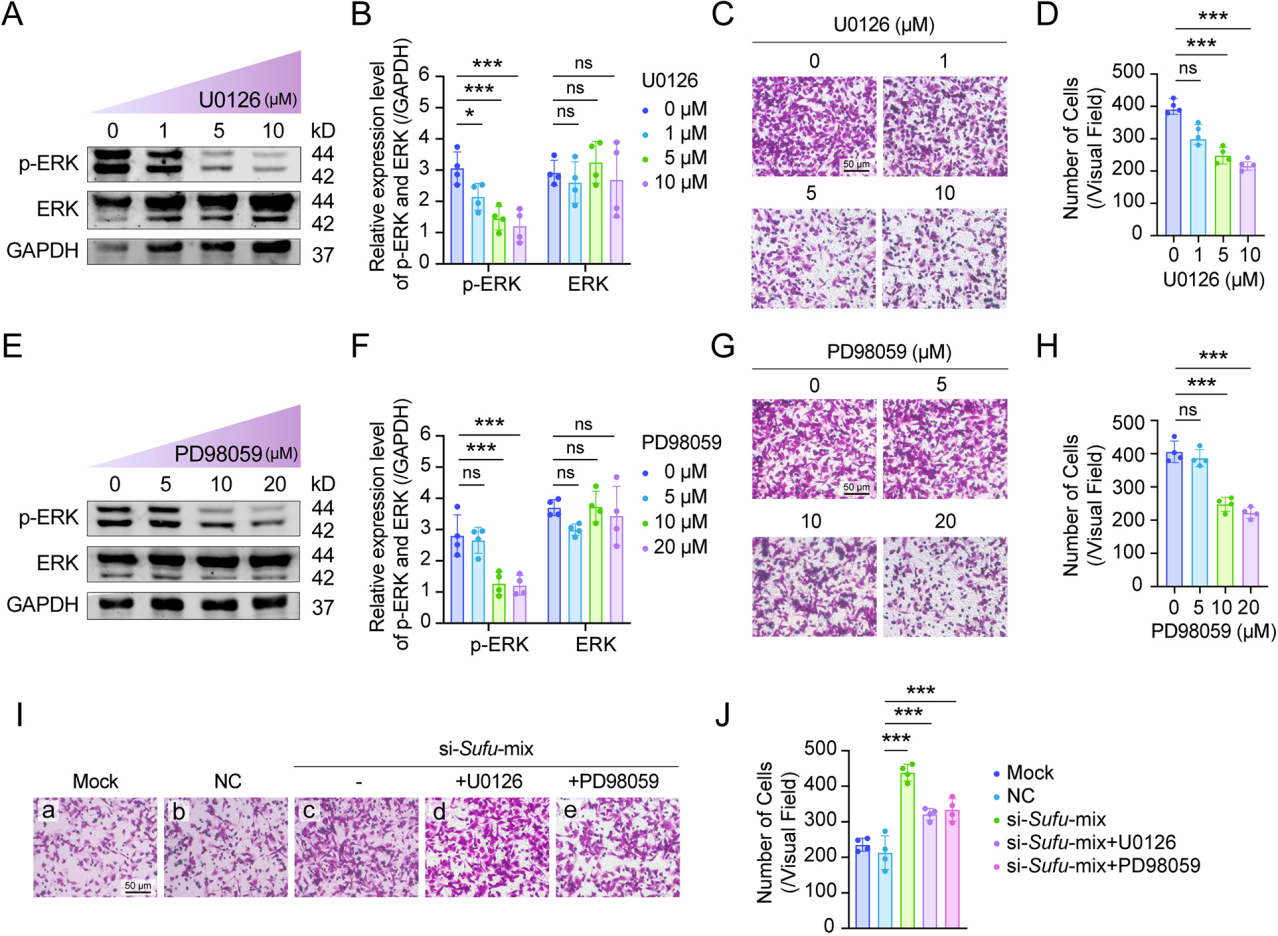
**MAPK inhibitors partially rescue the si-*Sufu*-mediated increase in melanoblast migration.** (A,B) Expression levels of phosphorylated (p)-ERK and ERK after treatment of melb-a cells with a concentration gradient of the MAPK inhibitor U0126 for 2 h (*n*=4). (C) Migration of melb-a cells as evaluated in a transwell migration assay after U0126 treatment at different concentrations (*n*=4 for each concentration). Note that p-ERK inhibition reduced melb-a cell migration. (D) Statistical analysis of the results based on B. (E,F) Expression levels of p-ERK and ERK after treatment of melb-a cells with a concentration gradient of the MAPK inhibitor PD98059 for 2 h (*n*=4). (G) Migration of melb-a cells as evaluated in a transwell migration assay after PD98059 treatment at different concentrations. Note that inhibition of p-ERK reduced melb-a cell migration. (H) Statistical analysis of the results based on G (*n*=4). (I) Migration of melb-a cells as evaluated after mock or control siRNA transfection or after the combined treatment with a mix of si-*Sufu*-1 and si-*Sufu*-2 (si-*Sufu*-mix), alone or in combination with U0126 or PD98059. (J) Statistical analysis of the results in I (*n*=4). Note that compared to treatment with si-*Sufu*-mix alone, treatment with si-*Sufu*-mix and either MAPK inhibitor partially inhibits melb-a cell migration. *P<0.05, ***P<0.001 (one-way ANOVA).

To confirm that SUFU indeed regulates melanoblast migration by modulating MAPK signaling, we blocked MAPK activity by using specific inhibitors *in vitro*. First, we verified the inhibitory efficiency of U0126 on p-ERK of MAPK signaling after treatment of melb-a cells with a concentration gradient of inhibitor for 2 h. As shown in Fig. 5A and B, the protein expression levels of p-ERK were significantly decreased with increasing concentrations of U0126, without changing the ERK levels. As a result, the migration of melb-a cells was decreased in a transwell migration assay (Fig. 5C,D). A second MAPK signaling inhibitor, PD98059, gave similar results (Fig. 5E-H). These data indicate that, *in vitro*, melanoblast migration can be inhibited by reducing MAPK signaling.

We reasoned that, if the above results were true, then the simultaneous downregulation of *Sufu* and inhibition of p-ERK activity should rescue the effect of selective *Sufu* downregulation. Hence, we applied the MAPK inhibitors U0126 or PD98059 to si-*Sufu*-1- and si-*Sufu*-2-treated melb-a cells and assayed them along with appropriate controls in the transwell assay. As shown in Fig. 5I and J, the combination of si-*Sufu* with either of the two inhibitors reduced the migration of melb-a cells compared to that of si-*Sufu* singly transfected cells, although not to the full extent of the migration seen with mock or control siRNA-transfected cells. Nevertheless, the results suggest that SUFU-mediated regulation of MAPK signaling in the melanocyte lineage is an important mechanism controlling the migratory behavior of melanoblasts.

### SUFU affects melanoblast migration by regulating MAPK signaling via GLI3-R

**Fig. 6.**
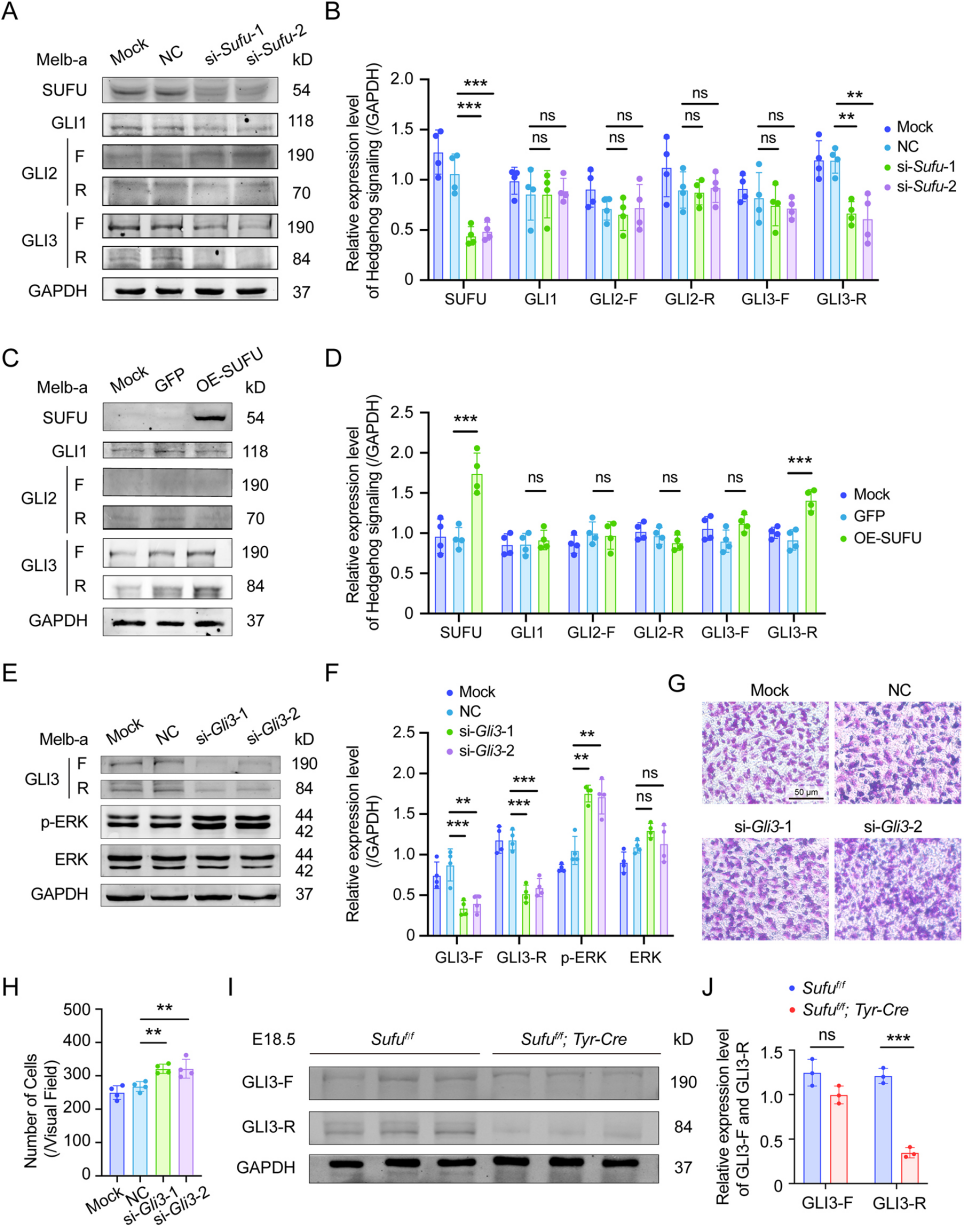
**GLI3 regulates the migratory capacity of melanoblasts via MAPK signaling.** (A) The expression levels of SUFU, GLI1, GLI2 and GLI3 in melb-a cells after mock, si-NC, si-*Sufu-1* or si-*Sufu-2* transfection. Note that GLI3-R was downregulated after interfering with *Sufu* (*n*=4). (B) Statistical analysis of protein expression based on the results in A. (C) Expression levels of SUFU, GLI1, GLI2 and GLI3 in melb-a cells after infection with mock, lentivirus-GFP and lentivirus-SUFU (OE-SUFU). Note that GLI3-R was upregulated after overexpressing SUFU (*n*=4). (D) Statistical analysis based on the results in C. (E) Expression levels of GLI3, p-ERK and ERK in melb-a cells after mock, si-NC, si-*Gli3*-1 or si-*Gli3*-2 transfection. Note that p-ERK was upregulated after interfering with *Gli3* (*n*=4). (F) Statistical analysis based on the results in E. (G) Migratory capacity was evaluated with a transwell migration assay after si-*Gli3* transfection. Note that the knockdown of *Gli3* promoted melb-a cell migration (*n*=4). (H) Statistical analysis based on the results in G. (I) Expression levels of GLI3-F and GLI3-R in the iris and ciliary body (rich in melanoblasts) of E18.5 *Sufu^f/f^* and *Sufu*-cKO embryos. Expression of GLI3-R was significantly decreased in the iris and ciliary body of *Sufu*-cKO embryos compared to that in controls. (*n*=4). (J). Statistical analysis of protein expression based on the results in I. **P<0.01, ***P<0.001; ns, no significant difference (two groups, unpaired two-tailed Student's *t*-test; multiple groups, one-way ANOVA).

To further address the regulatory mechanism between SUFU and MAPK signaling, we examined the protein expression levels of GLI1, GLI2 and GLI3, the transcriptional effectors of Hh signaling that are capable of directly binding SUFU (Cheng and Yue, 2008). As already mentioned, GLI1 comes in one form, whereas GLI2 and GLI3 each come as full-length (F) or truncated (R) forms. Western blots showed that, in melb-a cells, the expression levels of GLI1, GLI2-F and GLI2-R were low and were not altered significantly after *Sufu* knockdown. However, the expression levels of both GLI3-F and GLI3-R were high in melb-a cells, and GLI3-R was downregulated after si-*Sufu* transfection (Fig. 6A,B). Overexpression of SUFU led to an increase in the expression of GLI3-R, but there were no significant changes in the expression levels of the other GLI proteins and forms (Fig. 6C,D).

The above results suggested that, because at least GLI3-R levels were inversely correlated with p-ERK levels after SUFU knockdown, GLI3-R might be involved in p-ERK regulation. Hence, we modulated GLI3 levels by using si-*Gli3* in the absence of SUFU downregulation and examined the expression of p-ERK. As shown in Fig. 6E and F, the expression of p-ERK was slightly upregulated after si-*Gli3* transfection. As expected, transwell migration assays showed that si-*Gli*3 treatment promoted migration, albeit mildly (Fig. 6G,H). To confirm the above results, we examined ocular melanoblasts from the iris and ciliary body of E18.5 control and *Sufu*-cKO embryos. As shown in Fig. 6I and J, the expression of GLI3-R was significantly downregulated in melanoblasts of *Sufu*-cKO eyes compared to those of control eyes. These results suggest that SUFU modulates MAPK signaling by modulating p-ERK via GLI3-R and, in this way, regulates the migratory capacity of eye melanoblasts.

## DISCUSSION

Here, we demonstrate in mice that the melanocyte-specific knockout of a regulator of Hh signaling, SUFU, surprisingly creates a prominent phenotype only in melanocytes of the eye, and there only in a melanocyte subpopulation, those at the ciliary margin and the developing iris and not those in the choroid. Moreover, the melanocyte abnormality is associated with anterior segment dysgenesis and is apparently related to aberrant melanocyte migration and not proliferation. This latter observation is surprising as the melanocyte phenotype is mediated by an increase in p-ERK, which is part of the MAPK signaling pathway predominantly known for its role in increasing cell proliferation. These results lead to three major interrelated questions. What might be the mechanisms behind the region-specific effects of alterations in Hh signaling? Does the fact that the migration phenotype can be mimicked in a melanoblast cell line *in vitro* allow one to conclude that it is exclusively melanocyte autonomous? And what might be the reasons behind the fact that melanocyte migration rather than proliferation is affected? Answers to these questions will hopefully help us gain insights into the pathogenesis of anterior segment dysgeneses as seen in humans and point to potential therapeutic strategies.

The first question can be approached by considering that melanocytes come in clearly distinct subpopulations. Our results indicated that *Sufu* deficiency led to a prominent phenotype only in melanocytes of the developing iris, whereas there were no differences in melanocyte distribution in skin, ears, tails and paws between *Sufu^f/f^* and *Sufu*-cKO mice. In addition, there was no notable difference in epilation-induced hair follicular melanogenesis between *Sufu^f/f^* and *Sufu*-cKO mice. Generally speaking, there are neuroepithelium-derived melanocytes (RPE cells, see further below) and NC-derived ones, which are divided into cutaneous and extracutaneous types (Cichorek et al., 2013), the latter including the choroidal and uveal melanocytes of the anterior segment. Despite their common feature of synthesizing melanin, there are prominent differences between these populations. Cutaneous melanocytes, for instance, transfer their melanin-containing melanosomes to keratinocytes, whereas extracutaneous melanocytes or RPE cells do not. The different populations also depend on, or at least respond to, different signaling pathways: RPE cells respond, for instance, to WNT and BMP signaling, while cutaneous melanocytes respond to WNT, KIT and EDNRB signaling, as well as Agouti signaling protein and alpha-MSH; uveal melanocytes do not respond to alpha-MSH (Li et al., 2006), are less dependent on KIT signaling, but depend on EDNRB signaling (Aoki et al., 2009) and respond to WNT signaling. In mice homozygous for the *Kit* allele *Kit^V620A^*, melanocytes are absent in hair follicles but present in the iris and choroid (Aoki et al., 2009). In mice homozygous for *Kit^Wps^*, cutaneous melanocytes are deficient, but those in the choroid are only reduced in number (Li et al., 2020). Furthermore, malignancies (melanomas) derived from the two groups of cells are also different. They differ, for instance, in the oncogenic driver mutations, mainly alterations in MAPK signaling in cutaneous melanoma and alterations in GNAQ/GNA11 signaling or the BRCA1-associated protein BAP1 in uveal melanomas (Harbour et al., 2010; Phelps et al., 2022; Van Raamsdonk et al., 2009). They also differ in the major sites of metastases and in the response to therapies. Moreover, the critical melanocyte transcription factor MITF is essential for the development and maintenance of cutaneous melanomas but acts as a tumor suppressor in uveal melanoma (Phelps et al., 2022). WNT signaling has further complexities. For instance, WNT5A binding to either one of two receptors, FZD2 or FZD5, leads to the degradation of β-catenin, but binding to FZD4 and the co-receptor LRP6 leads to stabilization of β-catenin (Webster and Weeraratna, 2013).

Canonical WNT/β-catenin signaling plays a key role in melanocyte development (Larue and Delmas, 2006). Specifically, WNT proteins such as WNT1 and WNT3A, both of which have been implicated in the early events of specification of dorsal neural tube derivatives, are involved in the formation of melanocyte lineages. The absence of both WNT1 and WNT3A leads to a deficiency in NC-derived melanocytes (Ikeya et al., 1997), and murine melanocytes require WNT1 for both expansion and differentiation (Dunn et al., 2000). In addition, WNT activation drives melanocyte stem cell proliferation and differentiation into pigment-producing melanocytes during hair regeneration (Rabbani et al., 2011). It has been shown in several studies that *Sufu* negatively regulates WNT/β-catenin signaling in development and tumorigenesis (Coquenlorge et al., 2019; Meng et al., 2001; Yung et al., 2019). In this study, we found that loss of *Sufu* did not result in any changes to melanocytes during hair regeneration and did not alter the proliferation or pigmentation of ocular melanocytes, which might be regulated by the WNT signaling pathway. Whether canonical or non-canonical WNT signaling is involved in the regulation of abnormal ocular melanocyte migration in *Sufu*-CKO mice will require additional investigation.

It is unclear whether all of the above differences are cell intrinsic, i.e. exist regardless of the specific locations of the cells, or are cell extrinsic, i.e. are induced by the respective microenvironments. This brings us to the second of the above three questions: are the melanocyte phenotypes cell autonomous or not? The microenvironments in which the different melanocyte subpopulations are found are definitely distinct. Skin melanocytes are in intimate contact with keratinocytes, which influence their behavior, but keratinocytes are absent in the anterior segment of the eye or in the choroid. The choroid is a highly vascularized tissue replete with fibroblasts and immune cells (Nickla and Wallman, 2010), although fibroblasts are also present in the anterior segment. However, iridal fibroblasts in human express histamine receptors, but such receptors have not yet been reported in choroidal fibroblasts (Sharif and Crider, 2011). Moreover, melanocytes of the anterior segment are in closer proximity to the CMZ than are choroidal melanocytes, and the CMZ expresses specific growth factors (Kubo and Nakagawa, 2008; Marcucci et al., 2016) to support the local development of retinal stem cells and progenitor cells (Fischer et al., 2014; Zhao et al., 2002). Furthermore, the CMZ is partially controlled by Hh signaling, and a gain in Hh signaling in the chick leads to a smaller CMZ (Moshiri et al., 2005). Hence, one might speculate that there exists an Hh-influenced crosstalk between melanocytes and the CMZ and that altered Hh signaling in either melanocytes or the non-melanocytic part of the CMZ disrupts the proper formation of the anterior segment. In other words, even if SUFU knockout in melanocytes creates a melanocytic phenotype, one cannot exclude that this phenotype feeds back on the microenvironment, and an altered microenvironment may then, in turn, feedback on melanocytes and bias them to respond with, say, migration rather than proliferation. Nevertheless, to our knowledge, the selective absence of melanocytes, for instance in mice homozygous for viable *Kit* alleles, do not by themselves create anterior segment abnormalities.

So far we have not talked much about RPE cells, or more precisely about the anterior and iridal portions of the pigmented layer that extends the RPE. We recognize that, given our knockout strategy using a pigment gene promoter, RPE cells will lack SUFU function as much as NC-derived melanocytes do. However, at this point, we do not know whether this creates a relevant phenotype in these cells that may influence the uveal melanocytes and modify the anterior segment dysgenesis. These questions are currently under investigation by using RPE and NC melanocyte-specific knockout strategies.

Taken together, the above considerations show that RPE cells, and anterior segment, choroidal and cutaneous melanocytes are unquestionably different and could hence likewise differ with respect to the response to altered Hh signaling. To understand the nature of these subpopulation-specific differences in Hh signaling needs further exploration.

Let us finally consider the last of the three major questions mentioned above: why is migration and not proliferation affected? Of course, equating melanocyte mislocalization, which is what we actually documented, with altered migration is a short cut, as it would be possible, at least theoretically, that mislocalization is the result of abnormal retention at these sites and not of abnormal migration. This interpretation would require, though, that melanocytes would normally transit through these sites, for instance in the developing cornea, but there is absolutely no support for this assumption. Hence, altered migration remained the only reasonable hypothesis. To address the potential underlying mechanisms, we had to switch to an *in vitro* system, as the small number of anterior segment melanocytes prevented us from a direct *in vivo* analysis. Moreover, early attempts to establish cell lines from this cellular subpopulation were unsuccessful, and so we finally settled on the melb-a melanoblastic cell line. The cells of this line are admittedly not strictly related to uveal melanoblasts as they are originally derived from the integument (the melanocytes of which, see above, do not show a *Sufu* knockout phenotype). Nevertheless, to more closely reflect the growth conditions of uveal melanoblasts, we kept them in 12-O-tetradecanoylphorbol-13-acetate (TPA), which mimics EDNRB signaling, rather than in KIT ligand, which would be more appropriate for cutaneous melanoblasts. We found that, after SUFU knockdown, the TPA-treated melb-a cells recapitulated the presence of a migratory, and the absence of a proliferative, phenotype as seen in uveal melanocytes *in vivo*; hence, we deemed them appropriate to elucidate the underlying molecular alterations. First, we found that, in these cells, the Hh effectors GLI1 and GLI2 were expressed at very low levels, but GLI3 expression was easily visible in both its GLI3-F activator and GLI3-R repressor forms. SUFU knockdown led to the downregulation of GLI3-R but not GLI3-F, a result that we confirmed *in vivo* and that was consistent with previous results by Makino et al. (2015). This indicated that SUFU knockdown might shift the balance to an increase in Hh signaling. A SUFU–GLI3 axis has also been reported in regulating embryo patterning (i.e. not specifically in melanocytes) although with slightly different results. For instance, both GLI3-F and GLI3-R were found to be reduced in *Sufu* mutants (Jia et al., 2009), and, in a different study, loss of *Sufu* led to the destabilization of GLI3-F, although not GLI3-R (Wang et al., 2010). Our experiments in melb-a cells showed that GLI3 knockdown (thus reducing both repressor and activator forms) led to an increase in the cells' migratory capacity, consistent with what we saw after knocking down *Sufu* and hence suggesting that the reduction in GLI3-R outweighs the effect of a similar reduction in GLI3-F. In any event, we also observed an increase in p-ERK associated with the GLI3-R reduction. This is not unlike what can be seen in melanoma cells, in which an increase in p-ERK can lead to increased cell migration (Satyamoorthy et al., 2003). Conversely, the downregulation of ERK signaling with appropriate inhibitors led to a reduction in the SUFU knockdown-mediated increase in migration. Nevertheless, one should keep in mind that the modulation of p-ERK via GLI3-R after manipulation of SUFU levels may be just one of many molecular changes influencing melanoblast migration.

The above findings could also have medical implications because the phenotype of *Sufu*-cKO shares some similarities with ocular melanocytosis. Ocular melanocytosis is a congenital periocular pigmentary condition consisting of excess melanocytes within the periocular skin, uvea, sclera and other areas (Jager et al., 2020; Plateroti et al., 2017; Shields et al., 2013). Ocular melanocytosis is one of the risk factors for ocular melanoma (Plateroti et al., 2017). Recently, a cohort study revealed that the ratio of patients with ocular melanocytosis who developed uveal melanoma was 1 in 400 (as opposed to ∼1 in 200,000 people without melanocytosis) (Singh et al., 2011). Also, patients with melanocytosis who developed uveal melanoma have a higher risk for metastasis, and their metastases are more invasive than those of ocular melanoma patients without melanocytosis (Shields et al., 2013). Although the potential underlying mechanisms remain unknown, one suspects a link to mutations affecting NC cells (Abdolrahimzadeh et al., 2023; Benson and Rennie, 1992), with the possibility that such mutations may affect the migration of NC-derived melanocytes (Maniar et al., 2019; Plateroti et al., 2017). Hence, our *Sufu*-cKO mouse model could provide insights into the pathogenesis of this disorder.

**Fig. 7.**
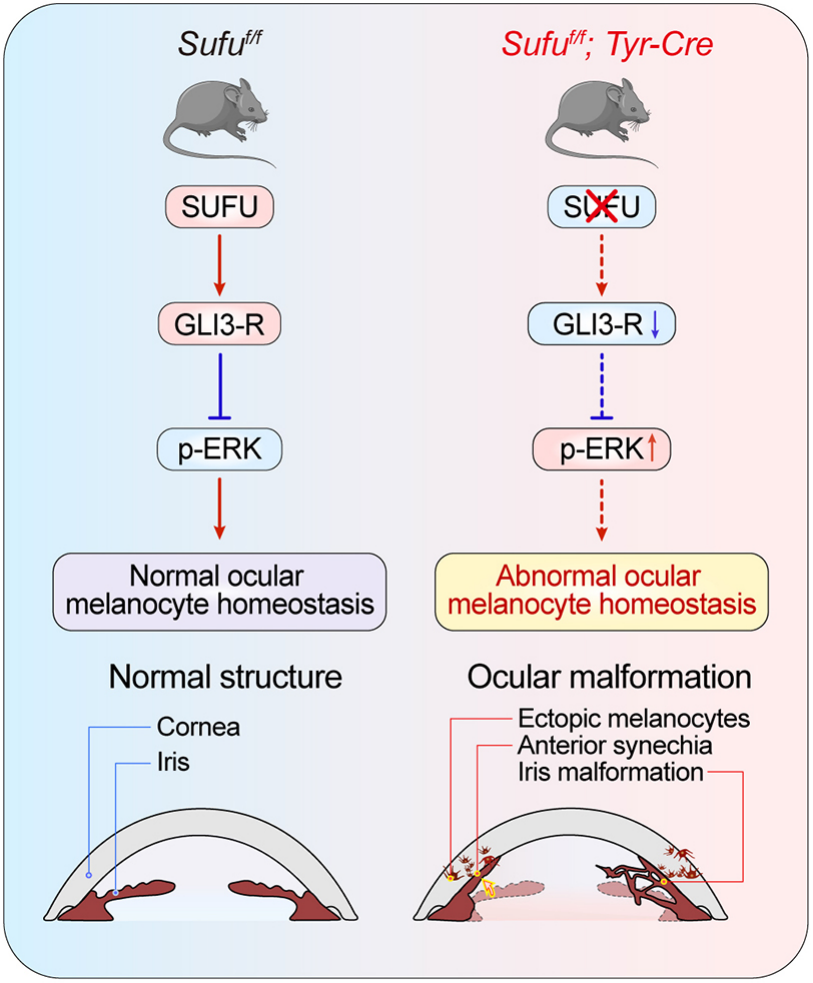
**Diagrammatic summary of the regulation of ocular melanocyte homeostasis by SUFU.** Left: SUFU maintains homeostasis of ocular melanocyte lineage through GLI3-R and p-ERK, resulting in normal melanocyte homeostasis and proper development of anterior segment structures in developing eyes. Right: loss of *Sufu* in the ocular melanocyte lineage results in the downregulation of GLI3-R and upregulation of p-ERK, which disrupts ocular melanocyte homeostasis and leads to ectopic melanocytes and abnormal anterior segment structures in developing eyes.

In summary, we have demonstrated that *Sufu* is crucial for maintaining melanocyte homeostasis, and its disruption leads to ectopic melanoblast migration in the anterior segment of the eyes during development. Aberrant ectopic ocular melanoblasts apparently migrate out from their proper locations, leading to anterior segment malformations and visual function impairment (Fig. 7). These findings contribute to our understanding of normal melanocyte homeostasis and the distinct roles of SUFU in subpopulations of ocular and cutaneous melanocytes, and could also help us understand the pathogenesis of melanocyte-related diseases in the human eye.

## MATERIALS AND METHODS

### Mouse strains

*Sufu^f/f^* mice and *Tyr-Cre* mice have been reported previously (Colombo et al., 2007; Li et al., 2017b, 2015). They were crossed to eventually generate *Sufu^f/f^; Tyr-Cre* (*Sufu*-cKO) mice on a C57Bl/6 background. *Sufu*-cKO mice were bred with *Sufu^f/f^* mice to obtain *Sufu^f/f^* control littermates as well as *Sufu*-cKO. Mice were kept on a 12-h/12-h light/dark cycle with food and water given *ad libitum*. Both sexes of mice were used for the experiments. All animal experimental procedures were performed in accordance with the Association for Research in Vision and Ophthalmology (ARVO) statement of the Use of Animals and the approved guidelines of the Wenzhou Medical University Institutional Animal Care and Use Committee (permit number wydw2022-0323).

### Genotyping by PCR and knockdown of *Sufu* by siRNA

All mice were genotyped by PCR analysis of genomic DNA extracted from tails or embryonic tissues using the primers listed in Table S1. PCR reactions were carried out by standard protocols.

Specific siRNA sequences and controls were designed and synthesized by GenePharma (Shanghai, China), as indicated in Table S2. Transfection was carried out at 60-70% confluency. Transfection of melb-a cells was carried out using a LipoJet Transfection Kit (SignaGen Laboratories, SL100468), according to the manufacturer's instructions.

### Histology and immunofluorescent staining

Isolated mouse eyeballs, whole embryos and other histological samples (including skin, ear, tail and paw) were fixed in a mixture of formaldehyde: ethyl alcohol: ddH_2_O: acetic acid glacial (1:4:4:1) at room temperature for 24 h. After gradient alcohol dehydration, the tissues were embedded in paraffin in a sagittal direction. The tissues were sliced into sections of 5 μm thickness and stained with H&E according to previous standard protocols (Chen et al., 2019).

For immunofluorescence, mouse eyeballs or whole embryos were fixed in 4% paraformaldehyde (PFA; Sigma-Aldrich, 158127) for 2 h, and the tissues were soaked in 30% sucrose in PBS at 4°C for 6-8 h. The tissue was then placed into Optimal Cutting Temperature Compound (Sakura, 4583) in a sagittal direction, frozen in liquid nitrogen, and sectioned at 10 μm thickness. Sections mounted on glass slides were incubated in 0.4% Triton X-100 (Sigma-Aldrich, T8787) for 10 min and blocked with bovine serum albumin (Sigma-Aldrich) for 1 h at room temperature. Slides were then incubated with primary antibodies overnight at 4°C. The following antibodies were used alone or in combination: anti-MITF (1:200; a gift from Dr Heinz Arnheiter, National Institutes of Health, Bethesda, MD, USA) and anti-Ki67 (1:200; Abcam, ab279653). After washing three times with 1× PBS for 5 min/time, slides were incubated with the corresponding host secondary antibodies (Alexa Fluor^®^, Invitrogen) for 1 h at room temperature. After washing, nuclei were counterstained with 4′,6-diamidino-2-phenylindole (DAPI; Beyotime Biotechnology, China; 1:1000 from a 1 mg/ml stock solution) and ProLong Gold Antifade medium (Invitrogen, P36930). Immunofluorescence images were obtained using a confocal microscope (LSM880 NLO with AiryScan, Carl Zeiss).

In each subgroup, four eyes were collected for sectioning and staining. For each eye sample, three separate, non-continuous sections were taken to count the positive signals in the iris ciliary body and then averaged as a data point in the statistical plot. A total of 12 slices (from four different samples) were counted for each subgroup.

### Epilation induction

Epilation was performed as described previously (Li et al., 2017a). Briefly, hairs of P21 mice were epilated using hair removal wax strips (Mayllie, Guangzhou, China) under ketamine and xylazine anesthesia to induce entry into anagen and lead to synchronized hair regeneration. After 3, 5 or 8 days, the wounding area (1 cm^2^ area of skin) was excised for histological analysis. Sample collection days of hair melanogenesis stages were determined according to comprehensive guidelines (Slominski et al., 1991, 1994, 1996).

### TUNEL staining

TUNEL staining was performed using a One-Step TUNNEL Assay Kit (Roche, 11684795910) as previously described (Chen et al., 2019). Briefly, ocular tissues were fixed with 4% PFA, embedded and sectioned. Then the sectioned slides were rinsed with PBS, and permeabilized with 0.4% Triton X-100 to obtain the fragmented DNA of apoptotic cells. Next, TUNEL detection solution was added to each tissue sample on sections, and the samples were incubated for 1 h at 37°C in a dark environment. Finally, the tissue sections were rinsed again with PBS and co-stained with DAPI.

### Western blot analysis

Western blotting was carried out as described previously (Chen et al., 2019). Briefly, proteins of tissues or melb-a cells were extracted using a radio immunoprecipitation assay buffer with 1 mM phenylmethanesulfonyl fluoride, phosphatase inhibitors and protease inhibitors. The concentration of each specimen was determined using a BCA Protein Quantitation Kit (Beyotime, Biotechnology), and the samples were separated with SDS-PAGE and transferred to nitrocellulose filter membranes. The membranes were blocked with 5% non-fat powdered milk (Beyotime Biotechnology) and then incubated with primary antibodies overnight at 4°C. The following antibodies were used: anti-SUFU (1:1000; Abcam, ab28283), anti-TGF-β (1:1000; Cell Signaling Technology, 3711S), anti-p-SMAD2/3 (1:1000; Cell Signaling Technology, 8828), anti-SNAIL (1:1000; Cell Signaling Technology, 3879T), anti-BMP4 (1:1000, Abcam, ab39973), anti-p-SMAD1/5/8 (1:1000, Cell Signaling Technology, 9511), anti-p-ERK (1:1000; Cell Signaling Technology, 9101S) and anti-ERK (1:1000; Cell Signaling Technology, 9102). The following day, the membranes were incubated with the corresponding host secondary antibodies for 1 h at room temperature. Odyssey^®^ CLx Imager was used to detect the blots, and the bands were quantified using ImageJ software.

### Cell cultures

Mouse melanoblast (melb-a) cells (a gift from Dr Dorothy Bennett, St George's, University of London, London, UK) were cultured at 37°C in RPMI-1640 medium (Sigma-Aldrich, R7388). Initial culturing was done in RPMI-1640 medium supplemented with 10% fetal bovine serum (FBS; Gibco, 16000-044), sodium pyruvate (Gibco,11360070), sodium bicarbonate (Gibco, 25080094), basic fibroblast growth factor (bFGF; R&D Systems, 3139-FB-025) and stem cell factor (KIT ligand, also called SCF; R&D Systems, 455-MC). To adjust the culturing conditions to more closely reflect the growth conditions of ocular melanocytes, which do not respond to KIT ligand (Aoki et al., 2009) but are activated through EDNRB, we then replaced KIT ligand and bFGF with TPA, which activates PKC and thus reflects EDNRB signaling. Cells were cultured in an atmosphere containing 95% air and 5% CO_2_.

Primary melanocyte cultures were prepared as described previously (Chen et al., 2016). Briefly, the epidermis of P6 skin of mice was separated from the underlying dermis by 0.25% trypsin and 50 U dispase treatments (37°C, 3 h). The separated dermis was sheared into pieces, followed by 0.05% trypsin treatment (37°C, 30 min). After trypsin neutralization, cells were collected and cultured in Dulbecco's modified Eagle medium (DMEM)/F12 supplemented with 10% FBS, 100 nM TPA (Sigma-Aldrich) and 5 μM forskolin (Sigma-Aldrich). The primary melanocytes were expanded twice (1:3 splits), and then cultured in DMEM/F12 with 10% FBS, 100 nM TPA in the absence of forskolin.

### Lentivirus and infection

Infection of melb-a cells was carried out using lentivirus-SUFU (1.96×10^8^ TU/ml) (Baixi, Hangzhou, China) or GFP-lentivirus (2.19×10^8^ TU/ml) with 5 μg/ml polybrene. Stable cell lines were obtained by screening with puromycin (2 μg/ml).

### Cell proliferation and migration assays

For cell proliferation analyses, melb-a cells were measured by a Cell Counting Kit-8 detection kit (CCK-8; Beyotime Biotechnology). All groups of cells were seeded at a concentration of 5×10^3^ cells per well in 96-well plates. All experiments were carried out in triplicate at 12, 24, 48, 72, 96 and 120 h after seeding. CCK-8 solution was applied at 10 μl per well, followed by 4 h incubation at 37°C. Absorbance values of all wells were then determined at 450 nm in Multimode Microplate Reader SpectraMax M5 (Molecular Devices).

The analysis of melb-a cell migration was analyzed in a transwell migration assay. After being starved for 12 h, 2×10^5^ cells per well were seeded and cultured in the upper chamber with 200 μl RPMI-1640 medium in a 24-well plate (Corning, 3422; 8 μm pore). The bottom well contained 600 μl complete culture medium. After 24 h, the upper chamber was fixed with 4% PFA for 20 min at room temperature and then incubated with Crystal Violet (Sangon Biotech, Shanghai, China) for 20 min at room temperature. The cells in the upper chamber were then wiped off with a cotton swab, and the chamber was soaked in PBS. The cultures were photographed using a microscope (Zeiss, Axio Observer 3) to reveal the cells that had migrated to the bottom chamber. Each experiment was repeated at least three times.

### Statistical analysis

All statistical tests were performed using GraphPad Prism (Version 8). For comparison between two groups, unpaired two-tailed Student's *t*-test was applied. For multiple comparisons, a one-way analysis of variance (ANOVA) with Tukey's post hoc test was used to assess statistical significance. Data are shown as mean±s.d. Error bars reflect independent experiments. *P*<0.05 was considered statistically significant.

## Supplementary Material

10.1242/dmm.050210_sup1 Supplementary information

## First Person

This article has an associated First Person interview with the first author of the paper.
