# Supplementary material for: Disrupting Hedgehog signaling in melanocytes by SUFU knockout leads to ocular melanocytosis and anterior segment malformation
Source: Dis Model Mech. 2023 Aug 29;16(8):dmm050210. doi: 10.1242/dmm.050210 (PMC10481947; doi:10.1242/dmm.050210)
Supplement: Supplementary information [file dmm-16-050210-s1.pdf]

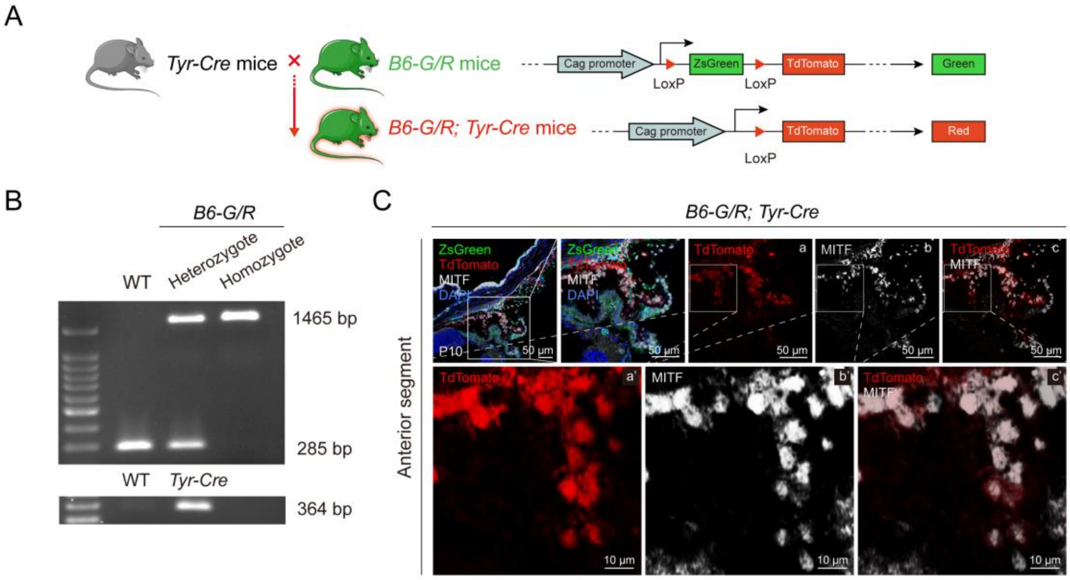

Fig. S1. Expression of *Tyr-Cre* in ocular melanocytes. (A) Schematic drawing of the *B6-G/R; Tyr-Cre* construct and the generation of a transgenic mouse. *ZsGreen* and *TdTomato* (driven by the promoter of *Cag*) are inserted into *B6-G/R* mice. **(B)** Gel electrophoresis of PCR reaction products was obtained using primers B6-G/R 5'-arm, B6-G/R Wild type, and Tyr-Cre (Table S1). Genomic DNA was isolated from tails from wildtype, *B6-G/R* heterozygote, and *B6-G/R* homozygote mice. **(C)** Representative immunostaining images of TdTomato (red) and MITF (white) of P10 *B6-G/R; Tyr-Cre* eyes. Note that the majority of melanocytes (MITF positive) showed red TdTomato fluorescence, indicating CRE expression. Images of a', b', and c' were enlarged images corresponding to images of a, b, and c.

Disease Models & Mechanisms • Supplementary information

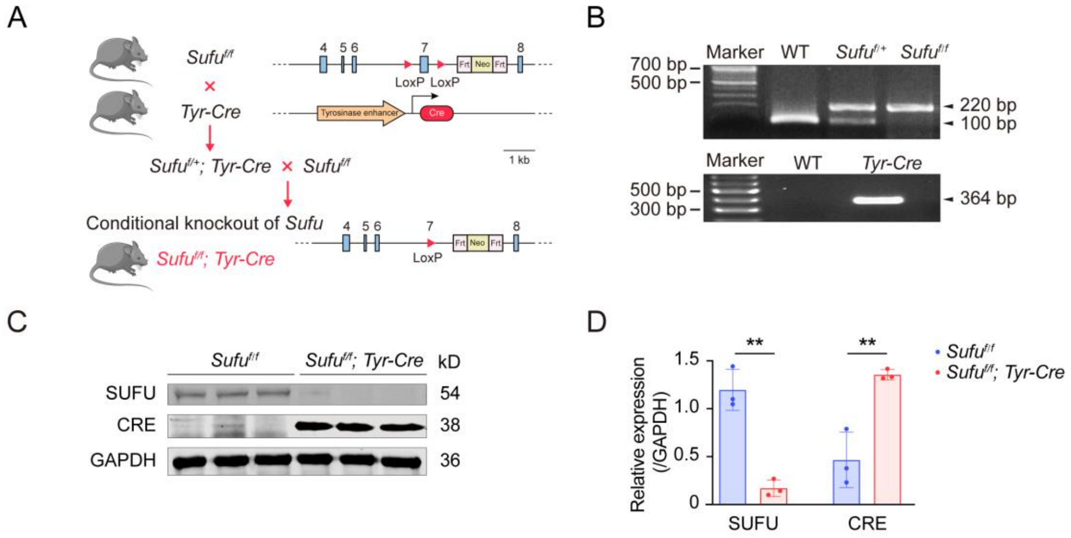

Fig. S2. Establishment of melanocyte-specific *Sufu* knockout mice. (A) Schematic drawing of the *Sufu*-cKO mouse before and after Cre-mediated recombination. **(B)** Gel electrophoresis of PCR reaction products obtained using primers *Sufu* and *Tyr-Cre* (Table S1). Genomic DNA was isolated from tails from wildtype, *Sufu*$^{f/f}$, and *Sufu*$^{f/f}$-cKO mice. **(C)** Western blot analysis shows the protein levels of SUFU and CRE in purified skin melanocytes from postnatal day 6 (P6) mice (n = 4). **(D)** Statistical analysis of western blot results based on (C). Bp: base pair. **$P< 0.01$, ***$P< 0.001$.

Disease Models & Mechanisms • Supplementary information

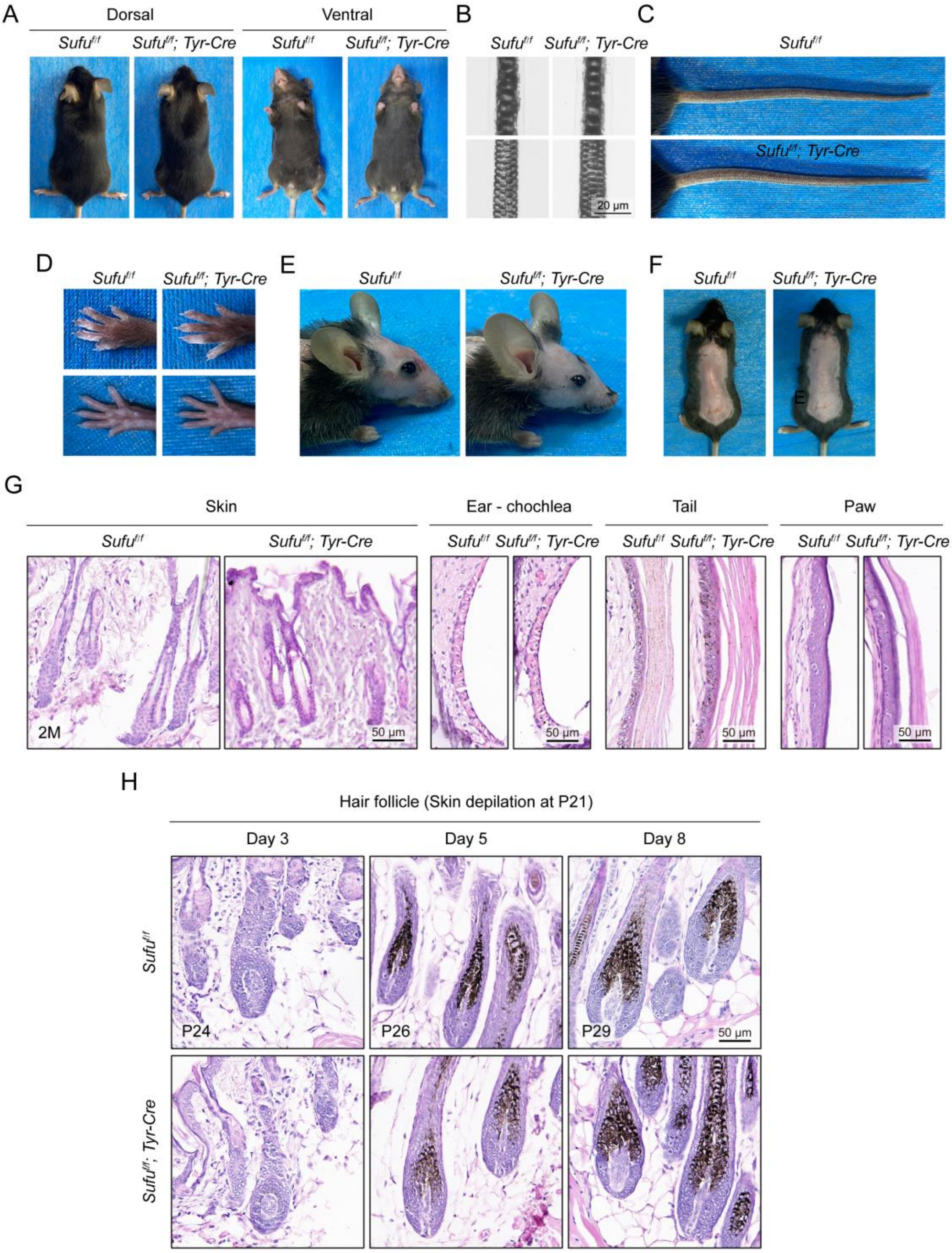

Fig. S3. Loss of *Sufu* in melanocyte lineage does not affect pigmentation and melanocytes of skin and hair follicles. (A) Images of the whole dorsal and ventral hair in 2-month-old *Sufu*$^{f/f}$ and *Sufu*-cKO mice. **(B)** Images of the hair pigmentation in 2-month-old *Sufu*$^{f/f}$ and *Sufu*-cKO mice. Scale bar: 20 μm. **(C)** Images of the tail of 2-month-old *Sufu*$^{f/f}$ and *Sufu*-cKO mice. **(D)** Images of the paw in 2-month-old *Sufu*$^{f/f}$

Disease Models & Mechanisms • Supplementary information

and *Sufu*-cKO mice. **(E)** Images of the epilated face skin in 2-month-old *Sufu*$^{f/f}$ and *Sufu*-cKO mice. **(F)** Images of the epilated back skin in *Sufu*$^{f/f}$ and *Sufu*-cKO mice. **(G)** Representative H&E staining images of the back skin, ear, tail, and paw in 2-month-old *Sufu*$^{f/f}$ and *Sufu*-cKO mice. Note that there were no significant differences in melanocytes distribution of skin, ear, tail, and paw between those mice with the different genotypes. **(H)** Representative H&E staining images of the hair follicles after skin depilation at P21 in *Sufu*$^{f/f}$ and *Sufu*-cKO mice. Note that there was no significant difference in epilation-induced hair follicular melanogenesis at day 3 (P24), day 5 (P26), and day 8 (P29) between the mice with the different genotypes.

Disease Models & Mechanisms • Supplementary information

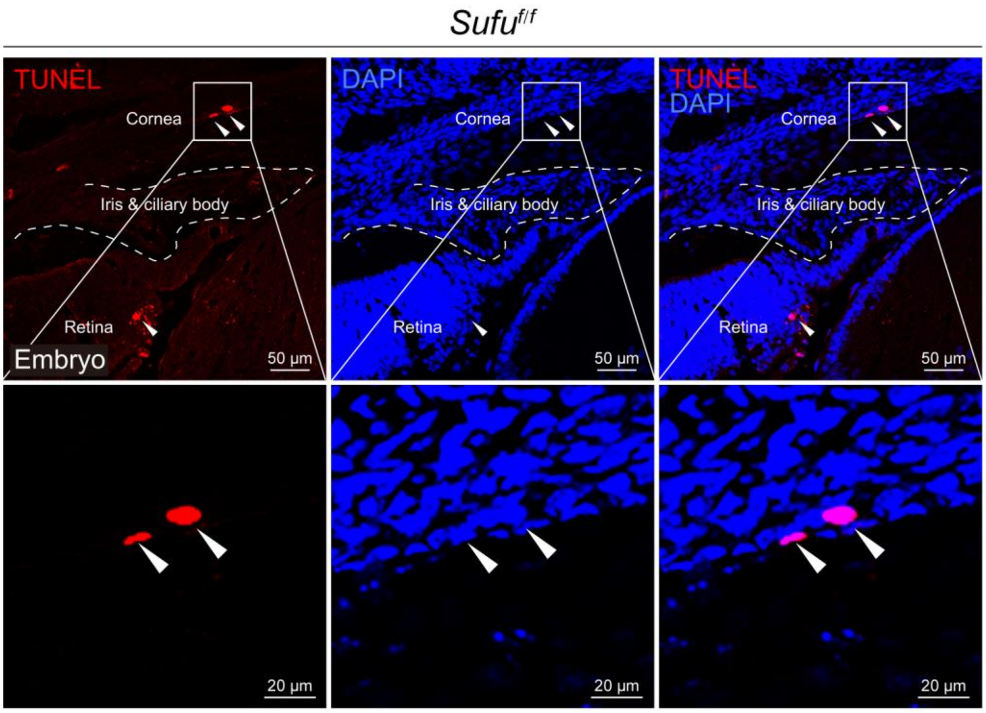

Fig. S4. TUNEL staining in the eyes of *Sufu*$^{f/f}$ embryos. The TUNEL-positive signal can be detected in the iris and ciliary body area of E18.5 embryos of the indicated genotypes, while a small amount of TUNEL-positive signals can be detected in the surrounding tissues such as the retina and cornea with a nuclear staining.

Disease Models & Mechanisms • Supplementary information

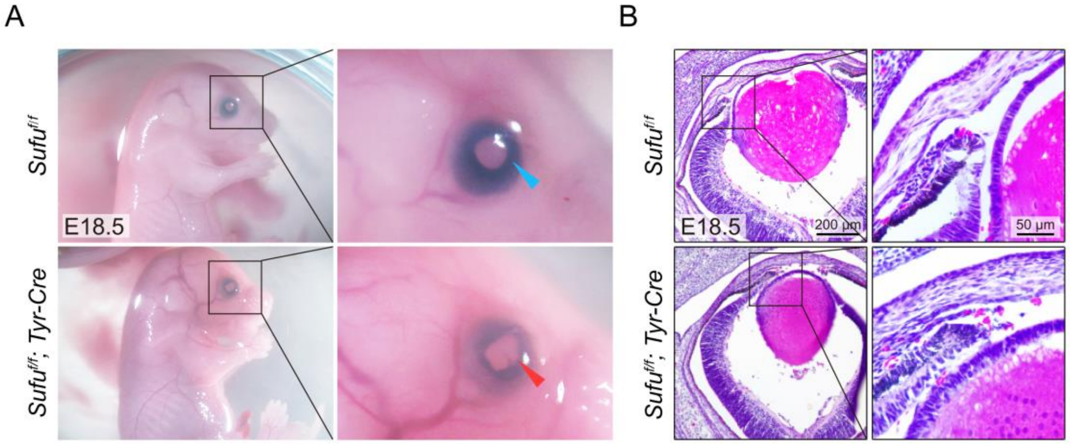

Fig. S5. Loss of *Sufu* in melanocyte lineage causes partial adhesion of the iris to the cornea in developing eyes of *Sufu*-cKO mice. (A) Representative images of E18.5 *Sufu*$^{f/f}$ and *Sufu*-cKO embryo showing iris malformation (blue arrow indicating the round and smooth pupil of *Sufu*$^{f/f}$ embryo; red arrow indicating an irregularly shaped pupil and iris malformation of *Sufu*-cKO embryo). **(B)** Representative histological H&E staining images of E18.5 *Sufu*$^{f/f}$ and *Sufu*-cKO embryos.

Table S1. Genotyping primers.

Allele	Sequence	Product size (bp)
B6-G/R 5'-arm	Forward: 5'-ATGCCCACCAAAGTCATCAGTGTAG-3'<br>Reverse: 5'-AGGCGGGCCATTTACCGTAAGTTA-3'	Wild type: 0<br>Targeted: 1465
B6-G/R Wild type	Forward: 5'-GGGCAGTCTGGTACTTCCAAGCT-3'<br>Reverse: 5'-ATATCCCCTTGTTCCCTTTCTGC-3'	Wild type: 285<br>Targeted: 0
Tyr-Cre	Forward: 5'-CAGCAGACACCAAGGAAACA-3'<br>Reverse: 5'-GCCAGGACCAAGAAGTGAGA-3'	364
Sufu	Forward: 5'-GTCTTGTCTCCTCCATCAGC-3'<br>Reverse: 5'-CAGGAATGAGCCTTGGACAC-3'	Wild type: 100<br>loxP: 220

Table S2. Si-RNA sequences.

Si-RNA	Sequence
Negative control	Sense: 5'-UUCUCCGAACGUGUCACGUTT-3'<br>Antisense: 5'-ACGUGACACGUGAGAATT-3'
Si-*Sufu*-1	Sense: 5'-GCCCUUUGGAUAACAGUGATT-3'<br>Antisense: 5'-UCACUGUUAUCCAAAGGGCTT-3'
Si-*Sufu*-2	Sense: 5'-GGAGAGGACUCGAGAUCAATT-3'<br>Antisense: 5'-UUGAUCUCGAGUCCUCUCCTT-3'
Si-*Gli3*-1	Sense: 5'-GGUGACCCUAUGCAUAAUATT-3'<br>Antisense: 5'-UAUUAUGCAUAGGGUCACCTT-3'
Si-*Gli3*-2	Sense: 5'-GCAGCCUCAAUAGCUUUAATT-3'<br>Antisense: 5'-UUAAAGCUAUUGAGGCUGCTT-3'

Disease Models & Mechanisms • Supplementary information
